# A central-peripheral approach to gait rehabilitation in chronic acquired brain injury: robotic training and motor imagery

**DOI:** 10.3389/fresc.2026.1874725

**Published:** 2026-07-31

**Authors:** Alessandro Arnaudo, Eugenio Scaliti, Francesco Damosso, Federica Lovera, Roberto Rebaudengo, Katiuscia Sacco, Marina Zettin, Andrea Cavallo

**Affiliations:** 1 Deparment of Psychology, University of Turin, Turin, Italy; 2 Centro Puzzle, Turin, Italy

**Keywords:** acquired brain injury, dual-task gait, gait analysis, motor imagery, rehabilitation, robot-assisted gait training

## Abstract

**Background:**

Gait impairment is a major long-term consequence of acquired brain injury and remains difficult to treat in the chronic phase. Robot-assisted gait training can deliver high-intensity repetitive practice, but robotic assistance alone may not fully engage central motor processes; motor imagery may complement this limitation.

**Objective:**

The primary aim was to evaluate the feasibility of an integrated protocol combining robotic gait training with structured motor imagery in adults with chronic acquired brain injury; a secondary, exploratory, aim was to characterize preliminary clinical signals and kinematic parameters observed in associated with the protocol.

**Methods:**

Seven adults completed 12 sessions over 4 weeks (3 sessions/week, about 30 min of active robotic walking per session). Outcomes included gait kinematics at baseline, post-intervention, and 3-month follow-up, plus Berg Balance Scale and 6-Minute Walk Test at baseline and post-intervention. Gait variables were analysed during both single-task and dual-task walking.

**Results:**

Clinical outcomes improved from baseline to post-intervention (Berg Balance Scale and 6-Minute Walk Test). For gait outcomes, session effects indicated improvement over time in temporal efficiency and phase organization on the more-affected limb, and task type effects were observed in key spatiotemporal variables, with expected dual-task costs maintained.

**Conclusions:**

This preliminary study supports the feasibility of combining robotic gait training with motor imagery in chronic acquired brain injury and provides exploratory, hypothesis-generating signals of concurrent clinical and kinematic change, to be tested in future controlled studies.

## 1 Introduction

Gait impairment is one of the most impacting long-term consequences of acquired brain injury (ABI), with substantial effects on autonomy, participation, and quality of life. Even after the acute phase, many individuals continue to experience reduced walking speed, altered gait patterns, and increased risk of falls that limit their ability and independence in daily life (1–4). In chronic ABI, spontaneous neurological recovery tends to plateau, making this stage particularly appropriate for the evaluation of intervention-specific effects and for the testing of rehabilitation strategies aimed at promoting functional reorganization.

In the last decade, robot-assisted gait training (RAGT) has emerged as a promising approach to address gait impairment after neurological injury. One of the main advantages of using RAGT is the ability to provide high-intensity repetitive task training by allowing patients to engage in controlled locomotor exercise under safe and adjustable conditions. This is considered crucial for favoring neuroplastic changes and improving gait performance. However, despite its theoretical advantages, evidence on RAGT effectiveness remains heterogeneous across studies, likely due to variability in devices, protocols, and patient populations (3, 5, 6). Moreover, a potential limitation of purely robotic approaches is that they may not always sufficiently engage higher-level motor and cognitive processes, particularly when movement execution is partially assisted and externally guided.

Indeed, from a functional perspective, walking is not just a biomechanical activity but a complex cognitive-motor function that requires the integration of motor planning, sensory feedback, and executive control (7). This becomes especially evident in everyday situations, where individuals must often walk while performing concurrent cognitive tasks. After ABI, impairments in attention and executive functions can exacerbate gait instability under such dual-task conditions, increasing the risk of falls and limiting real-world mobility (4, 8–10). Therefore, rehabilitation approaches that target not only motor execution but also the cognitive and representational aspects of movement may provide additional benefits.

In this context, motor imagery (MI) may represent a valuable complementary strategy. MI refers to the deliberate mental simulation of a movement or an action without actually executing it (11–14). Neurophysiological and neuroimaging studies have shown that MI engages motor-related brain networks that largely overlap with those involved in actual movement execution, including premotor, parietal, supplementary motor, and cerebellar regions (15–17). Such functional equivalence has led to the hypothesis that MI can activate internal motor representations and support motor re-learning processes, even in the absence of physical movement. Importantly, MI is not a unitary construct: it includes both visual imagery, involving the mental representation of movement from an external or third-person perspective, and kinesthetic imagery, which emphasizes the internal sensory experience of movement, such as proprioceptive and muscular sensations (14, 18). The latter is considered particularly relevant for motor rehabilitation, as it more directly engages sensorimotor representations associated with action execution (19).

From a cognitive perspective, by rehearsing movement sequences mentally, individuals may refine internal models of action, anticipate sensory consequences, and facilitate subsequent motor performance (20, 21). Consistent with this view, in the sport psychology literature, a growing body of evidence suggests that MI can improve motor outcomes when used as an adjunct to physical practice, with stronger effects observed when imagery is combined with actual movement training rather than delivered in isolation (22, 23). In gait rehabilitation, studies have shown that MI-based interventions can improve spatiotemporal gait parameters, such as step length and cadence, particularly in individuals with chronic neurological conditions, although findings across studies remain heterogeneous (19, 24, 25).

Taken together, these considerations suggest that combining MI with RAGT may offer a complementary central-peripheral approach to gait rehabilitation: robotic training provides repeated, structured sensorimotor input at the peripheral level, while MI enhances central motor engagement by reactivating internal representations of movement. Based on this rationale, the present study was conceived as a preliminary, hypothesis-generating feasibility study of an integrated robotic gait training protocol (REhabilitation HerO, REHO) combined with structured MI in individuals with chronic ABI. In addition to standard clinical outcomes, we examined gait kinematics under both single-task and dual-task conditions, in order to capture changes not only in motor performance but also in cognitive-motor integration. The primary aim of the study was thus to assess the feasibility of the combined RAGT + MI protocol in a single-arm design; the secondary, exploratory, aim was to characterize preliminary clinical signals and kinematic parameters in order to inform the design of subsequent controlled protocols.

## 2 Methods

### 2.1 Participants

This preliminary single-cohort study included seven adults (3 females, mean age of 49.8 ± 11.4 years) with chronic ABI recruited at a specialized neurorehabilitation center (Centro Puzzle, Turin, Italy). Participant-level characteristics are reported in Table 1. Inclusion criteria were: i) documented central neurological lesion (CT and/or MRI); ii) chronic stage (all ≥12 months post-injury) with residual gait impairment; iii) ability to maintain upright standing, (Berg Balance Scale, BBS ≥ 20); iv) sufficient cognitive-linguistic abilities to follow instructions (Montreal Cognitive Assessment, MoCA ≥ 21); v) sufficient motor imagery capacity to actively engage in imagery training, (Kinesthetic and Visual Imagery Questionnaire, KVIQ ≥ 60).

**Table 1:** Participant table. Participant age was calculated as the elapsed time, in years, between the T0 experiment date and the date of birth (assuming 365.25 days per year), and months from event was calculated as the elapsed time, in months, between the T0 experiment date and the ABI event date (assuming 30.44 days per month).

**Participants Table**								
**Patient ID**	**Sex**	**Age**	**ABI Etiology**	**More-Affected Hemisoma**	**Months from event**	**KVIQ Score**	**MoCA Score**	**Gait Aid Tested**
1	M	29.0	TBI	Right	46.1	80	24/30	Walker + Crutch
2	M	60.4	Ischemic Stroke	Right	27.3	88	29/30	Walker
3	F	49.7	MCA Aneurysm	Right	19.6	83	22/30	Crutch + No Aid
4	F	45.9	Hemorrhagic Stroke	Left	26.6	78	23/30	Walker
5	F	57.6	ICA Aneurysm	Right	43.6	82	27/30	Crutch + No Aid
6	M	44.6	Ischemic Stroke	Right	12.4	83	22/30	Walker
7	M	61.4	TBI	Left	336.0	81	22/30	Crutch

Exclusion criteria included severe musculoskeletal limitations incompatible with robotic gait training, unstable medical conditions, severe spasticity or clonus, and major cognitive-perceptual deficits compromising safety. All participants provided written informed consent. The study was conducted in accordance with the Declaration of Helsinki and was approved by the local ethics committee (protocol n. 0657504).

### 2.2 Study design and intervention

Participants underwent a structured intervention that combined RAGT with MI. The protocol consisted of 12 sessions over 4 weeks (3 sessions/week), with approximately 30 min of active robotic gait training per session.

RAGT was administered using the REHO system (REhabilitation HerO; Nimble Robotics: https://nimblerobotics.com/reho), an active bilateral lower-limb exoskeleton mounted on a ceiling rail with body-weight support, allowing overground walking with adjustable assistance at hip, knee, and ankle joints. Assistance levels and body-weight support were individualized and progressively adapted based on participant tolerance and performance.

Each session included setup procedures followed by repeated assisted walking bouts. To enhance sensorimotor integration, walking segments alternated between conditions emphasizing visual feedback (mirror-based) and proprioceptive/kinesthetic input (eyes closed). All participants completed the rehabilitation protocol, with the necessary individual customizations, without complications. More in-depth feasibility indicators were recorded for each participant and summarized in Supplementary Table S1.

### 2.3 Motor imagery protocol

MI was systematically integrated within robotic training sessions. During scheduled pauses (approximately every 2 min), participants performed brief MI blocks guided by prompts.

Imagery was performed with eyes closed and first-person perspective, alternating between kinesthetic and visual components. An example of kinesthetic imagery prompt was: “Imagine taking three steps forward while focusing on how your body weight shifts from one leg to the other during walking.” An example of visual imagery prompt was: “In the position where you stopped, can you say which leg is in front? Now imagine four steps forward from this position and indicate which leg will be in front at the end.”

Participants were instructed to mentally produce step sequences, starting from a few imagined steps and progressively increasing according to tolerance and imagery quality. Brief probe questions were used throughout sessions to verify engagement. The MI protocol was designed to align temporally and functionally with ongoing gait training in order to activate motor representations associated with walking.

A session-by-session overview of the MI protocol is provided in Supplementary Material (See Supplementary Table S2). MI sessions were administered by two staff members directly involved in the design and standardization of the protocol (a physiotherapist and a neuropsychologist). Patients' attendance was recorded for all sessions, and no participant missed a scheduled session. No additional formal fidelity-monitoring procedure (e.g., session audio-recording or independent checklist review) was implemented beyond adherence to the standardized protocol.

### 2.4 Clinical and gait assessment

Clinical outcomes included Berg Balance Scale (BBS), assessing functional balance (26), and 6-Minute Walk Test (6MWT), assessing walking endurance (27). Clinical measures were collected at baseline (T0) and post-intervention (T1).

Instrumented gait analysis was conducted at baseline (T0), post-intervention (T1), and at 3-month follow-up (T2), to evaluate changes in spatiotemporal and kinematic parameters. Clinical follow-up at T2 was not collected, so long-term inferences are restricted to kinematic outcomes.

### 2.5 Kinematic protocol (gait analysis)

Gait data were collected using an optoelectronic motion-capture system (Vicon, 100 Hz sampling rate; https://www.vicon.com) with a standard lower-body model marker set (16 markers, 9.5 mm).

Participants performed overground walking in a 5-meter room under two conditions:

- Single-task (ST): walking at self-selected speed.
- Dual-task (DT): walking at self-selected speed while performing a concurrent auditory attentional task that required the detection and verbal signaling of consecutive identical letters within a randomized sequence.

Participants completed repeated walking trials according to tolerance (from 6 to 12 trials per condition), with rest breaks as needed. They walked either without assistance or with their habitual aid (walker, crutch, or no assistance), and this configuration was tracked for statistical modeling.

Raw kinematic data were preprocessed in MATLAB (https://www.mathworks.com) using custom scripts, low-pass filtered with a Butterworth filter (8 Hz cutoff). Walking data were then segmented into discrete gait strides (heel-strike to heel-strike) using custom-developed MATLAB software.

Analyses focused on spatiotemporal variables and selected kinematic parameters from the more-affected limb. Kinematic outcomes included i) Cadence: number of steps per minute; ii) Stride Length: distance between two consecutive heel strikes of the same foot; iii) Stride Time: time between two consecutive heel strikes of the same foot; iv) Stance/Swing Phase: relative duration of stance and swing phases; v) Swing Velocity: velocity of the heel during the swing phase; vi) Ankle Flexion at Strike: ankle flexion angle at heel-touch; vii) Ankle Dorsi-Flexion Range of Motion (ROM). The single gait stride was adopted as the unit of analysis for all statistical models reported below.

### 2.6 Statistical analysis

Gait outcomes were analysed using generalized linear mixed models (GLMM), including Session (T0, T1, T2) and Task Type (ST, DT) as fixed effects. Random effects included Subject and Walking Aid (walker, crutch, or no assistance), to account for between-subject heterogeneity in gait performance and between different walking aids. Formally, for each gait outcome Y, the original model took the form:

Y∼Session×TaskType+(1|Subject)+(1|WalkingAid)

where Session and Task Type were fixed effects, and Subject and Walking Aid were modelled as random intercepts. Then, candidate models with main effects and Session × Task Type interaction were compared using Bayesian Information Criterion (BIC), and the best-fitting model was retained (28). In the presence of a significant main effect of Session, planned *post hoc* comparisons were conducted to compare T0 vs. T1 and T0 vs. T2. Pairwise contrasts were corrected with Holm-Bonferroni adjustment, and 95% confidence intervals of the difference between conditions were computed for the GLMM contrasts.

Clinical outcomes (BBS, 6MWT) were analysed using one-tailed paired Wilcoxon signed-rank tests (T0 vs. T1), in line with the *a priori* directional hypothesis of expected improvement from T0 to T1 in both BBS and 6MWT. Statistical significance was set at *p* < 0.05.

## 3 Results

### 3.1 Gait outcomes

**Figure 1:**
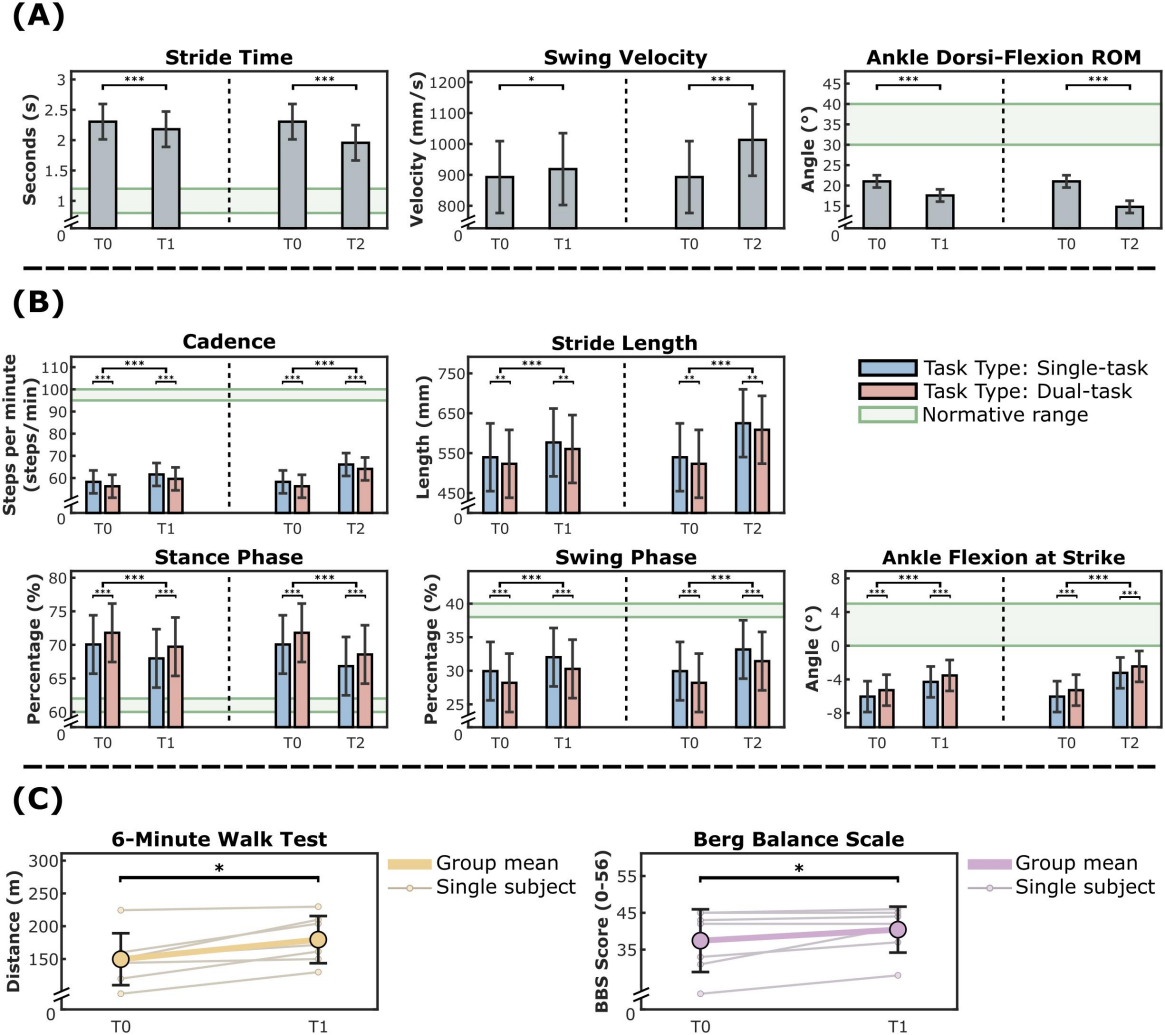
Clinical and gait outcomes across sessions (T0, T1, T2) and task conditions (ST, DT). **(A)** Gait variables with Session-only main effect from GLMM: stride time, swing velocity, and ankle dorsi-flexion range of motion (more-affected side). *post-hoc* contrasts are shown for T0-T1 and T0-T2. Horizontal shaded bands indicate normative reference ranges where available (stride time around 0.8–1.2s; ankle sagittal excursion around 30–40°), derived from healthy gait references. **(B)** Gait variables with both Session and Task Type main effects: cadence, stride length, stance phase, swing phase, and ankle flexion at foot strike (more-affected side). ST and DT trajectories are plotted separately; *post-hoc* contrasts are shown for T0-T1 and T0-T2, and normative reference bands are displayed for cadence (approximately 95–100 steps/min), stance-swing distribution (approximately 60%–40%), and ankle flexion angle at initial contact (approximately 0–5°) based on normative adult gait data. **(C)** Clinical outcomes (BBS and 6MWT) at T0 and T1. Data are shown as estimated marginal means ± SE at the population level, with overlaid individual trajectories.

The best-fitting models included main effects of Session and/or Task Type depending on the variable (Figures 1A,B). No significant Session × Task Type interaction was retained in the selected models.

Variables with Session main effect only (Figure 1A) were:

- **Stride Time**: mean ± SE: 2.30 ± 0.29 s (T0), 2.18 ± 0.29 s (T1), 1.96 ± 0.29 s (T2); Session main effect: *p* < 0.001; Session contrasts T0-T1 *p* < 0.001, T0-T2 *p* < 0.001. 95% CI for T0-T1: [−0.18 −0.07]; T0-T2: [−0.40 −0.29].
- **Swing Velocity**: mean ± SE: 892.91 ± 116.28 mm/s (T0), 918.70 ± 116.29 mm/s (T1), 1,013.22 ± 116.17 mm/s (T2); Session main effect: *p* < 0.001; Session contrasts T0-T1 *p* = 0.011, T0-T2 *p* < 0.001. 95% CI for T0-T1: [4.85 46.72]; T0-T2: [98.94 141.69].
- **Ankle Dorsi-Flexion ROM**: mean ± SE: 21.00 ± 1.51° (T0), 17.55 ± 1.51° (T1), 14.76 ± 1.51° (T2); Session main effect: *p* < 0.001; Session contrasts T0-T1 *p* < 0.001, T0-T2 *p* < 0.001. 95% CI for T0-T1: [−4.18 −2.72]; T0-T2: [−6.98 −5.50].

Variables with both Session and Task Type main effects (Figure 1B) were:

- **Cadence**: mean ± SE: ST = 58.26 ± 5.17 (T0), 61.60 ± 5.17 (T1), 66.09 ± 5.17 steps/min (T2); DT = 56.28 ± 5.18 (T0), 59.62 ± 5.18 (T1), 64.11 ± 5.17 steps/min (T2). Session main effect: *p* < 0.001; Session contrasts T0-T1 *p* < 0.001, T0-T2 *p* < 0.001; Task Type main effect: *p* < 0.001. 95% CI for Session T0-T1: [2.40 4.28]; T0-T2: [6.87 8.79]; ST vs. DT: [−2.63 −1.33].
- **Stride Length**: mean ± SE: ST = 539.66 ± 85.00 (T0), 576.80 ± 85.01 (T1), 625.00 ± 84.94 mm (T2); DT = 523.26 ± 85.07 (T0), 560.40 ± 85.07 (T1), 608.60 ± 85.00 mm (T2). Session main effect: *p* < 0.001; Session contrasts T0-T1 *p* < 0.001, T0-T2 *p* < 0.001; Task Type main effect: *p* = 0.0096. 95% CI for Session T0-T1: [23.45 50.84]; T0-T2: [71.36 99.33]; ST vs. DT: [−25.90 −6.90].
- **Stance Phase**: mean Stance ± SE: ST = 70.06 ± 4.35 (T0), 67.98 ± 4.35 (T1), 66.83 ± 4.35% (T2); DT = 71.80 ± 4.36 (T0), 69.72 ± 4.36 (T1), 68.57 ± 4.35% (T2). Session main effect: *p* < 0.001; Session contrasts T0-T1 *p* < 0.001, T0-T2 *p* < 0.001; Task Type main effect: *p* < 0.001. 95% CI for Session T0-T1: [−2.83 −1.32]; T0-T2: [−4.00 −2.46]; ST vs. DT: [1.22 2.27].
- **Swing Phase**: mean Swing ± SE: ST = 29.94 ± 4.35 (T0), 32.02 ± 4.35 (T1), 33.17 ± 4.35% (T2); DT = 28.20 ± 4.36 (T0), 30.28 ± 4.36 (T1), 31.43 ± 4.35% (T2). Session main effect: *p* < 0.001; Session contrasts T0-T1 *p* < 0.001, T0-T2 *p* < 0.001; Task Type main effect: *p* < 0.001. 95% CI for Session T0-T1: [1.32 2.83]; T0-T2: [2.46 4.00]; ST vs. DT: [−2.27 −1.22].
- **Ankle Flexion at Strike**: mean ± SE: ST = −6.05 ± 1.83 (T0), −4.30 ± 1.83 (T1), −3.22 ± 1.83° (T2); DT = −5.28 ± 1.84 (T0), −3.53 ± 1.84 (T1), −2.46 ± 1.83° (T2). Session main effect: *p* < 0.001; Session contrasts T0-T1 *p* < 0.001, T0-T2 *p* < 0.001; Task Type main effect: *p* < 0.001. 95% CI for Session T0-T1: [1.24 2.26]; T0-T2: [2.31 3.34]; ST vs. DT: [0.42 1.11].

### 3.2 Clinical outcomes

Participant-level scores for the BBS and 6MWT at T0 and T1 are reported in Table 2. Clinical outcomes improved from baseline to post-intervention. Specifically, BBS increased from 37.43 ± 3.22 at T0 to 40.43 ± 2.36 at T1 (*p* = 0.03; Figure 1C), and 6MWT increased from 149.69 ± 14.95 m at T0 to 179.54 ± 13.60 m at T1 (*p* = 0.01; Figure 1C).

**Table 2:** Clinical assessment. Individual scores for the 6MWT and BBS performed at T0 (baseline) and T1 (post-intervention).

**Clinical Assessment**				
**Patient ID**	**BBS Score (T0)**	**BBS Score (T1)**	**6MWT (T0)**	**6MWT (T1)**
1	31/56	41/56	152.4 m	171.6 m
2	33/56	37/56	160 m	204 m
3	43/56	44/56	144 m	150 m
4	23/56	28/56	120 m	161.4 m
5	45/56	46/56	224.4 m	229.8 m
6	42/56	42/56	97 m	130 m
7	45/56	45/56	150 m	210 m

## 4 Discussion

This study explored the feasibility of an integrated RAGT + MI protocol as its primary aim, and characterized preliminary, exploratory efficacy signals in individuals with chronic acquired brain injury as a secondary aim. From a feasibility standpoint, all seven participants completed the full 12-session protocol with complete adherence and no adverse events, and tolerance to the combined RAGT + MI protocol was rated as medium-to-high across participants (Supplementary Table S1). This supports the practical viability of integrating structured MI within a RAGT session without additional burden on session duration or patient tolerance, addressing a precondition for testing efficacy signals in future controlled studies.

The protocol was also associated with improvements in both clinical and gait-related outcomes. Balance improved by 3 points on the BBS and walking endurance increased by approximately 30 m on the 6MWT. At the kinematic level, significant longitudinal effects were observed for stride time, swing velocity, cadence, stride length, stance-swing phase distribution, and ankle flexion at foot strike, indicating reorganization of gait cycle structure on the more-affected side. Importantly, these kinematic improvements were present under both single-task and dual-task conditions and were largely maintained at 3-month follow-up.

From a clinical perspective, the magnitude of improvement in the 6MWT is consistent with the minimal clinically important difference (MCID) reported for chronic neurological populations (29, 30). The BBS change can also be evaluated in comparison to the available MCID literature. The observed 3-point increase exceeds most of the MID estimates reported for chronic stroke populations using ROC-based and distribution-based methods (31), which supports describing the change as clinically meaningful. Moreover, clinical improvement is coherent with the kinematic reorganization observed in gait analysis: reduction in stride time and increase in swing velocity indicate improved temporal coordination of the gait cycle. The concurrent changes in cadence, stride length, and phase distribution suggest a shift toward more efficient walking, in the expected direction of healthy gait organization (32–35).

A key aspect of the study is the integration of MI within RAGT as an active form of action simulation. This central-peripheral coupling is relevant because, on one side, RAGT provides structured peripheral input and movement consistency, while on the other side, MI may increase intentional motor engagement and support consolidation of more efficient movement strategies (24, 25, 33). In the present protocol, the emphasis on first-person kinesthetic imagery was specifically designed to engage sensorimotor representations linked to proprioceptive timing and phase control (12, 18). Although the absence of a control group prevents causal attribution, the temporal reorganization observed here, particularly the reduction in stride time and the increase in swing velocity, is in line with what would be expected from an intervention that strengthens internal timing representations.

### 4.1 Gait quality and phase organization

Kinematic analyses focused on the more-affected side for each participant revealed coherent changes across temporal, spatial, and phase-related dimensions: shorter stride time, higher swing velocity, and progressive stance-swing redistribution, together supporting a shift toward more efficient cycle organization. This pattern is relevant because stance-swing phase distribution, swing velocity, and ankle position at initial contact jointly reflect phase efficiency, dynamic stability, and quality of foot placement, all aspects that go beyond simple speed gains (34, 36). Moreover, the simultaneous increase in stride length and cadence suggests that participants did not improve through isolated compensatory strategies alone, but through broader reorganization of step execution (32, 33, 37–39).

The reduction in ankle sagittal ROM should be interpreted cautiously. Session contrasts were significant for T0-T1 and T1-T2, but the direction of change was opposite to a simple expectation of larger joint excursion. In the context of concurrent global gait gains, this pattern may indicate reduced passive or compensatory ankle excursion and a tighter, more selective active control of the joint, with ankle motion constrained to the range functionally necessary for stepping. This interpretation remains tentative, but still consistent with hemiparetic gait literature describing walking as a dynamic mixture of primary deficits and adaptive compensations, where functional improvement can involve kinematic reorganization rather than uniform amplitude expansion across all joint variables (1).

### 4.2 Dual-Task performance

In the present study, dual-task walking showed reduced performance compared to single-task walking across most kinematic variables, confirming persistence of cognitive-motor interference in chronic ABI. However, the absence of a Session × Task Type interaction indicates that longitudinal improvements were present under both conditions. This suggests that observed gains extend to cognitively demanding contexts that better approximate everyday mobility. One parameter-specific exception emerged: ankle flexion at strike showed a relatively more favourable pattern in dual-task, with dual-task values appearing more favourable than single-task values at all sessions. A plausible interpretation is that dual-task load produced the expected decrement in global spatiotemporal variables, while for this fine-grained event-level parameter it may have reduced explicit over-control and favoured a more automatic execution mode, as described in attention-focus research showing that skill-focused monitoring can worsen well-learned sensorimotor performance whereas divided-attention contexts can preserve or even improve it in specific conditions (40). At the same time, because dual-task paradigms generally worsen gait in neurological populations, this finding should be treated as parameter-specific and exploratory rather than generalized (41). Lastly, the cognitive task was intentionally used as an attentional load manipulation rather than as a cognitive performance measure, and accuracy was not formally quantified. The presence of a significant Task Type main effect across multiple independent kinematic variables (with a consistent pattern of gait slowing, stride length reduction, and more cautious spatiotemporal adjustments under dual-task conditions) provides indirect behavioral evidence that the concurrent task was effectively engaging attentional resources during walking.

### 4.3 Limitations and future directions

The current preliminary study is explicitly limited by the lack of a parallel control group, which prevents causal attribution of observed gains to MI specifically, to RAGT alone, or to their combination. Accordingly, this study provides proof-of-concept and early efficacy signals rather than conclusive evidence.

As a single-arm, pre-post design, the study is also vulnerable to practice and learning effects across repeated sessions and assessments. While the convergence of change across multiple independent kinematic parameters, beyond clinical scales, argues against a purely artifactual explanation, these alternative explanations cannot be ruled out and should be considered when interpreting the current results.

Some aspects of protocol delivery and reporting were also not formally tracked. MI sessions were delivered by two dedicated staff members following a standardized prompt script, but no formal fidelity-monitoring procedure (e.g., session recording, independent checklist review) was implemented beyond this standardization. Outcome assessment was not blinded: both clinical evaluators and the kinematic analyst were aware of session timing (T0/T1/T2). For clinical scales, this represents a source of potential bias common to single-arm designs. For kinematic outcomes, however, this risk is mitigated by the fact that gait variables were extracted through a standardized, algorithmic MATLAB pipeline based on objective spatiotemporal and kinematic definitions, rather than through subjective clinical judgment.

The small sample size and etiological heterogeneity further limit generalizability, and individual differences in motor imagery capacity leave open the question of whether imagery ability modulates response to this type of intervention. A REHO-only control cohort is currently being planned to directly estimate the incremental contribution of MI.

In conclusion, this preliminary study supports the feasibility of integrating structured MI within RAGT in chronic ABI and provides a coherent preliminary signal across clinical and kinematic outcomes. Temporal gait-cycle reorganization, including shorter stride time, higher swing velocity, and redistribution of stance-swing phase, is consistent with improved motor organization rather than isolated compensatory gains, and is observable under both single-task and dual-task conditions. In a chronic population, where spontaneous recovery is typically limited, this framework appears to be particularly suitable for evaluating treatment-specific effects. The next controlled phase, a RAGT-only cohort with harmonized outcomes, will be essential to quantify the specific contribution of MI and to move toward a reproducible multimodal protocol for broader clinical implementation.

## Data Availability

The raw data supporting the conclusions of this article will be made available by the authors, without undue reservation.
