# Supplementary material for: A central-peripheral approach to gait rehabilitation in chronic acquired brain injury: robotic training and motor imagery
Source: Front Rehabil Sci. 2026 Jul 31;7:1874725. doi: 10.3389/fresc.2026.1874725 (PMC13473374; doi:10.3389/fresc.2026.1874725)
Supplement: Supplementary file 1 [file Supplementaryfile1.docx]

A central-peripheral approach to gait rehabilitation in chronic acquired brain injury: Robotic training and motor imagery

Alessandro Arnaudo^1^, Eugenio Scaliti^1^, Francesco Damosso^2^, Federica Lovera^2^, Roberto Rebaudengo^2^, Katiuscia Sacco^1^, Marina Zettin^1,2+^, Andrea Cavallo^1*+^

^1^Deparment of Psychology, University of Turin, Italy

^2^Centro Puzzle, Turin, Italy

^+^ Senior Author

**SUPPLEMENTARY MATERIAL**

*Corresponding author:*

Andrea Cavallo - ORCID: 0000-0002-3717-3760

Department of Psychology - University of Turin, Via Verdi 10, 10124, Turin (IT)

phone number: +390116703074

e-mail: [andrea.cavallo@unito.it](mailto:andrea.cavallo@unito.it)

**SUPPLEMENTARY MATERIAL**

**Table S1.** Protocol Feasibility Report. Individual-level protocol feasibility measures for each participant. Note that the duration of each MI block tended to increase over the course of the intervention, as the therapist progressively increased the complexity of the administered questions across sessions (see Table S2 for session-by-session details of the MI protocol).

| **Protocol Feasibility Report** | | | | |
| --- | --- | --- | --- | --- |
| **Patient ID** | **Adverse Events** | **Observed Tolerance (low – medium – high)** | **Average setup and session duration** | **MI Components** |
| 1 | NO | HIGH | SETUP: 20 min  REHO + MI: 35 min | Number of imagery sessions completed: 10/10  Imagery time: 15 min |
| 2 | NO | HIGH | SETUP: 20 min  REHO + MI: 30 min | Number of imagery sessions completed: 10/10  Imagery time: 10 min |
| 3 | NO | MEDIUM | SETUP: 20 min  REHO + MI: 30 min | Number of imagery sessions completed: 10/10  Imagery time: 10 min |
| 4 | NO | MEDIUM | SETUP: 20 min  REHO + MI: 30 min | Number of imagery sessions completed: 10/10  Imagery time: 10 min |
| 5 | NO | MEDIUM | SETUP: 20 min  REHO + MI: 30 min | Number of imagery sessions completed: 10/10  Imagery time: 10 min |
| 6 | NO | HIGH | SETUP: 20 min  REHO + MI: 35 min | Number of imagery sessions completed: 10/10  Imagery time: 15 min |
| 7 | NO | MEDIUM | SETUP: 20 min  REHO + MI: 35 min | Number of imagery sessions completed: 10/10  Imagery time: 15 min |

**Table S2.** Detailed description of the Motor Imagery protocol.

| **Motor Imagery Protocol** | |
| --- | --- |
| **Week 1** | |
| **Session 1** | |
| **Key aspects** | **Questions** |
| - Explanation of treatment procedures and goals. - Explanation of the cognitive functions involved. - Explanation of robotic device functioning. - Focus on proprioceptive aspects related to movement. - Focus first on the left leg, then the right leg, then both together. - Motor imagery exercises focused on alternating legs during walking. | - "In the position where you stopped, can you tell me which leg is in front?" - "Now we will focus on your left leg. Can you tell me whether your left leg was moving forward or backward?" - "Try to imagine taking 4 steps forward from your current position. At the end of the 4 steps, which leg will be in front?" |
| **Session 2** | |
| **Key aspects** | **Questions** |
| - Recap of the previous session. - Focus on proprioceptive aspects related to foot movement during walking. - Motor imagery exercises focused on ankle and foot movement. | - "What position are your feet in now? Are both feet fully in contact with the ground?" - "Try to imagine 3 steps forward, focusing on ankle movement during walking. Which part of the foot contacts the floor first?" - "During the next two minutes, focus on the movement that the device is producing at your feet." |
| **Session 3** | |
| **Key aspects** | **Questions** |
| - Recap of previously covered aspects. - Focus on proprioceptive aspects related to knee movement during walking. - Motor imagery exercises focused on knee movement. | - "At this moment, is your left leg extended or flexed? And your right leg?" - "When you start walking again, which leg will need to flex/extend?" - "Imagine taking 3 steps forward; at the end, will your left leg be extended or flexed?" |
| **Week 2** | |
| **Session 4** | |
| **Key aspects** | **Questions** |
| - Recap of previously covered aspects. - Active perception of body-weight distribution on the legs. - Imagery exercises focused on weight shift during walking. | - "At this moment, which leg is bearing more of your body weight?" - "Try to imagine 3 steps forward, focusing on body-weight transfer from one leg to the other during walking." - "During the next two minutes, pay attention to how your body weight shifts from one leg to the other while walking with REHO." |
| **Session 5** | |
| **Key aspects** | **Questions** |
| - Recap of previously covered aspects. - Perception and imagery of knee/foot interaction. | - "What position are your feet in right now? And your knees?" - "When your heel contacts the ground, is your leg extended or flexed? And when you lift the foot?" - "Imagine 2 steps forward, paying attention to knee and ankle movement." |
| **Session 6** | |
| **Key aspects** | **Questions** |
| - Recap of previously covered aspects. - Perception and imagery of knee/foot/body-weight interaction. | - "Describe the position you are currently in, paying attention to all the aspects we have discussed so far." - "Imagine taking 4 steps forward. What movements do your knees make? And your feet? At what moment in the step do you shift weight from one leg to the other?" |
| **Week 3** | |
| **Session 7** | |
| **Key aspects** | **Questions** |
| - Recap of previously covered aspects. - Bring attention to the origin of step-related movement. - Focus and imagery exercises on pelvis and hip movement during walking. | - "When we walk, where does movement start from?" - "Imagine taking 4 steps forward. Can you focus on the movement of your hips?" |
| **Session 8** | |
| **Key aspects** | **Questions** |
| - Recap of previously covered aspects. - Questions focused on what happens when walking backward. | - "Imagine taking one step backward. Which part of the foot contacts the ground first? And what movement does the knee make?" - "Imagine taking 3 steps backward. At the end of the steps, which leg will be more forward?"  Note: These questions are also used to avoid a learning effect. |
| **Session 9** | |
| **Key aspects** | **Questions** |
| - Recap of previously covered aspects. - Bring attention to whole-body movement during walking. - Focus and imagery exercises on upper-body movement during walking. | - "When you walk, how does the upper part of your body move? How do your arms move?" - "Try to imagine taking 5 steps forward, focusing on all the movements you perform during walking." |
| **Week 4** | |
| **Session 10** | |
| **Key aspects** | **Questions** |
| - Recap of previously covered aspects. - Increased focus on aspects that were more difficult for the patient. - If working-memory capacity allows, increase the number of imagined steps while maintaining detailed imagery of each component of the step by recalling movements performed during REHO walking. | - "Try to imagine 6 steps forward. At the end, which leg will be in front? Remember: it is important to focus not only on leg alternation, but on all the movements you perform while walking." - "Imagine taking n steps, and try to keep the same rhythm you use when walking with the device, also during imagery." |
| **Session 11** | |
| **Key aspects** | **Questions** |
| - Recap and consolidation of previously covered aspects. | **/** |
| **Session 12** | |
| **Key aspects** | **Questions** |
| - Recap and consolidation of previously covered aspects. | **/** |
